# 940 Low-Dose Buprenorphine Initiation in Acute Burn Setting

**DOI:** 10.1093/jbcr/iraf019.471

**Published:** 2025-04-01

**Authors:** Mladen Nisavic, Jeremy Goverman, Matthew Supple

**Affiliations:** Massachusetts General Hospital; Massachusetts General Hospital; Massachusetts General Hospital

## Abstract

**Introduction:**

Opioid use disorder (OUD) is a significant cause of unintentional burn injury, and has been linked with higher 30-day readmission rates, higher total hospitalization costs and reduced length of stay in burn patients.

Medical treatment for OUD with buprenorphine (BUP) is the standard of care, and hospital setting presents an important moment to engage patients with treatment. Historically, BUP initiation in surgical setting has been avoided due to worries it may precipitate withdrawal or block conventional pain agents through partial agonist effect.

Low-dose buprenorphine initiation (LDBI) is a novel approach where low doses of BUP are administered with daily adjustments to facilitate controlled displacement of full agonist off the mu-receptor, thus minimizing the risk of precipitating withdrawal. Though this approach has been shown to be effective across a variety of medical settings, to our knowledge, there are no studies exploring potential feasibility and effectiveness of LBDI following acute burn injury.

**Methods:**

Following IRB approval, electronic medical records (EMR) were quarried over a 4-year period to identify all patients who received BUP while admitted to the burn unit. All records were reviewed to identify patients who received BUP consistent with LDBI. Data pertaining to full-agonist opioid use, psychiatric/addiction comorbidities, and complications related to LBDI was collected.

**Results:**

A total of 88 patients received BUP during the study period - of these, 14 received LBDI. Most patients (n = 12) completed the initiation readily without significant challenges. One patient noted minor pain issues related to LBDI, and 2 LBDI patients left self-directed; no other complications were noted. All patients received short-acting opioids or methadone; all doses were consistent with usual unit practices. Five LBDI patients were transitioned off full-agonist agents to BUP monotherapy on discharge, and 4 required short (5-7 day) bridging full agonist script.

**Conclusions:**

LDBI is a safe and feasible approach in acute burn setting, offering benefits to acute pain and withdrawal management and further benefit of reducing prescription opioid needs on discharge.

**Applicability of Research to Practice:**

Our workshop will review LBDI protocol within the acute burn setting and discuss adjunct analgesia approaches and strategies to manage anxiety or withdrawal concerns. Benefits to adequately managing pain and withdrawal concerns in burn patients with OUD are significant. Furthermore, transitioning patient onto evidence-backed treatment (BUP) on discharge leading to reduced opioid exposure (and thus reduced risk for relapse and associated complications) is a considerable benefit.

**Funding for the Study:**

N/A

